# Love and People With Intellectual Disabilities: A Meta‐Ethnography of Qualitative Research in the United Kingdom

**DOI:** 10.1111/jar.70132

**Published:** 2026-02-17

**Authors:** Rachel Forrester‐Jones, Manar Mustafa, Amy Randall

**Affiliations:** ^1^ School of Health Studies, Faculty of Health Sciences University of Western Ontario London Ontario Canada; ^2^ Department of Social & Policy Sciences University of Bath, Bath UK

**Keywords:** intellectual disabilities, intimacy, love, meta‐ethnography, romantic relationships

## Abstract

**Background:**

Love is part of human experience, yet people with intellectual disabilities face numerous barriers to expressing and engaging in loving relationships. Few UK studies have investigated this topic in depth.

**Methods:**

Eight databases were searched for articles published between 1998 and 2024. Article selection followed PRISMA and CASP guidelines. Included articles were subjected to thematic analysis.

**Results:**

Eight UK‐based studies met the inclusion criteria. These explored how individuals conceptualised love, their experiences of loving relationships, and factors influencing their romantic lives. Participants were able to articulate what love meant to them and how experiences of love linked to their identity, purpose, and well‐being. Barriers, including lack of privacy, control of carers and social isolation, were identified as hindering participants' pursuit of love.

**Conclusion:**

Policies need to facilitate inclusive practices that challenge societal and structural attitudes around love and romance for people with intellectual disabilities.

**Summary**

- For many individuals with intellectual disabilities, love and romantic relationships bring emotional fulfilment.
- We looked at studies that asked people with intellectual disabilities what they think about love and intimate relationships.
- Many barriers, including lack of privacy, societal stigma, and overprotective caregivers exist that limit people's ability to pursue and sustain romantic relationships. We call for policy and cultural changes that affirm their relational and emotional needs.

## 1 Introduction

Romantic love may be traced to the ancient Sumerians in 2500 bc and remains a contested concept (Forrester‐Jones et al. 2023). Most recently, Chen et al. (2024) found that undergraduates conceptualise romantic relationships through three core love factors: positive responsiveness (to needs), authentic connection, and a sense of stability, highlighting the multifaceted nature of love. In the field of intellectual disabilities, love has been defined as the desire for an attachment to another that is intimate and reciprocal (Bates et al. 2016b). ‘Intimacy’ in this context is defined in terms of relationships that go beyond the boundaries of being ‘just good friends’, emphasising ‘…closeness, understanding and loving, as well as kissing, touching, and sexual intimacy’ (White and Barnitt 2000, 271).

Adults with intellectual disabilities often express their desire for a long‐term romantic and loving sexual relationship (Bates 2019; Forrester‐Jones et al. 2002). Many say they would like to love and be loved by a person who is not paid to support them (Ward et al. 2010; McCarthy et al. 2022). In their meta‐synthesis of 16 qualitative studies about how people with intellectual disabilities viewed their sexual lives, Black and Kammes (2019) found that individuals desired friendship, intimacy and pleasure, as well as recognition of their sexual identity. However, policies as well as paid and family carers often thwarted intimate relationships. These practices resemble (Gomez's 2012) critique of the exclusion of romantic and erotic needs of people with intellectual disabilities:

Establishing and maintaining sexual and intimate relationships during an adult life is an expectation in most cultures, and yet, people with intellectual disability are sexually disenfranchised (2012, 241).

Historical attitudes labelled sexual relationships between people with intellectual disabilities as controversial and unpleasant (Bunyan et al. 1986) with some asserting that ‘…people with learning disabilities^1^ are sex crazed monsters’ (Landman 1994, 35) as cited in (White and Barnitt 2000, 271). Others characterised this population as asexual (Heyman 1995) necessitating segregation for their protection from sexual predators (Edgerton 1979). These views have largely dissipated and the advantages of fostering positive interpersonal relationships for physical and mental health, and eliminating social stigma is acknowledged. Evidence also indicates that staff and parents are assisting people with intellectual disabilities to establish loving relationships (Bates et al. 2020); (Bates et al. 2021) alongside the emergence of dating agencies (see McCarthy et al. 2020). In the United Kingdom (UK) these affirmative practices may have been shaped by legislation including the (Human Rights Act 1998), which enshrined rights for: dignity and respect in all aspects of individuals' care and support (Article 3), respect for private and family life (Article 8); to marry and start a family (Article 12) and protection from discrimination (Article 14). Valuing People (Department of Health 2001) and Valuing People Now (Department of Health 2009) also emphasised the need for health and social care services to challenge negative attitudes and improve services. Recommendations included providing accessible sex education. More recently, under the 2014 Care Act, local authorities must promote assessments and care plans that focus on achieving wellbeing outcomes including relationship aspirations.

Over time, increased opportunities have emerged for developing social relationships including supported employment (Forrester‐Jones et al. 2004), social media (White and Forrester‐Jones 2020) and dating agencies (McCarthy et al. 2020). Nevertheless, romantic relationships remain an area fraught with difficulties and frustrations for people with intellectual disabilities. Ward et al. (2010) interviewed 47 men and women with intellectual disabilities, finding that their romantic relationships mirrored those of people without intellectual disabilities, except that time spent together was more limited than preferred. (Hollomotz and the Speakup Committee 2009) studied privacy around sexual relationships in residential settings involving four focus groups with 15 self‐advocates. Participants discussed how institutional monitoring and lack of privacy restrained their quest for meaningful relationships. Further, (Abbott 2015) found that people with intellectual disabilities engaging in consenting same‐sex relationships experienced high levels of bullying and discrimination from paid carers and the public. Abbot argued that although individuals requested support to develop relationships with other Lesbian, Gay, Bisexual and Transgender men and women, the ‘gay scene’ was generally unwelcoming, and paid carers reluctantly viewed support for this life domain as part of their job role.

We carried out an initial scoping exercise using Google Scholar, Web of Science, SCOPUS and Campbell Collaborative. This revealed empirical studies concerning how people with intellectual disabilities experienced sexuality and sexual identity (Brown and McCann 2018), sexual intimacy (e.g., Craft 1994; Fish 2016; McCarthy 1999, 2014; Charitou et al. 2021); sexual and reproductive health, and starting a family (Baines et al. 2018); (Pérez‐Curiel et al. 2023), capacity to consent to sexual relationships (Murphy and O'Callaghan 2004), HIV prevention (Cambridge 1997), and sexual abuse (Thompson and Brown 1997). Harassment and discrimination faced by individuals identifying as LGBTQ^2^ (e.g., Bates 2020; Dinwoodie et al. 2020; English et al. 2018; Azzopardi Lane et al. 2019; Thompson et al. 2001) had also been investigated. However, we found few studies and no systematic reviews about how love was conceptualised or romantic relationships were experienced by adults with intellectual disabilities in the UK. Our purpose was to fill this gap.

Romantic relationship needs are more challenging to assess and address than physical needs (Amado 1993) which may account for the dearth of research in this area. The preponderance of a medicalized perspective (Oliver 2013) implying people with intellectual disabilities are unable and/or have little desire for romantic relationships, may also have impeded research and practice. Our review was informed by the social‐affirmation model (see Swain and French 2000; Malli and Forrester‐Jones 2025).

This theoretical framework affirms and embraces the differences people with intellectual disabilities have compared to the general population whilst also critically examining the limitations society places on them. Stigmatised views and prejudicial attitudes about people with intellectual disabilities lead to discriminatory behaviour at interpersonal, structural and systemic levels. By focusing less on individual limitations and more on societal obstacles, we hoped to highlight disparities in service responses to individuals' romantic needs thereby promoting change.

Given that love is inherently abstract and subjective, defining and measuring it presents challenges, especially when researching individuals with intellectual disabilities. Many individuals face communication and/or articulation difficulties and barriers that hinder their ability to convey these intricate emotions. However, qualitative research methods that provide time for researchers to build rapport and utilise adaptive communication styles can permit a deeper exploration and understanding of individuals' perspectives and experiences (Forrester‐Jones and Grant 1997); (Cambridge and Forrester‐Jones 2003). They also allow space for participants to define love on their own terms—fitting with the social‐affirmation model. We, therefore, chose to conduct a qualitative systematic review, focusing on the voices of individuals with intellectual disabilities rather than the perspectives of formal and informal carers, including parents.

## 2 Aims

Our systematic review aimed to find out how people with intellectual disabilities conceptualise love and romantic relationships. It was also our aim to investigate what mediates or hinders individuals' pursuit, development, and maintenance of loving relationships. As outlined above, our objective was to inform understandings and discourse around the topic and identify any research and practice gaps.

## 3 Method and Analysis

We ran our systematic review using ‘meta‐ethnography’ (Noblit and Hare 1988). Meta‐ethnography utilises induction and interpretation in order to represent descriptions and interpretations of qualitative data. It also reinterprets and builds upon overall study findings, generating new theories that may be tested by further research (Noblit and Hare 1988, 9–10). Noblit and Hare (1988, 26–29) proposed a seven‐step process for conducting meta‐ethnographies. Steps are changeable in terms of order and can be repeated. Although similar to other systematic review typologies, steps six and seven are differentiated since they move the review forward to a higher level of induction. Having decided on our topic (*Step 1*), we adapted Noblit and Hare's process as follows.

*Step 2*: Deciding on an inclusion criteria aimed to maximise breadth of understanding whilst ensuring that only the most relevant studies would be included (see Table 1).

**TABLE 1:** Inclusion and exclusion criteria.

Criteria	Included	Excluded
Participants	Individuals with intellectual and developmental disabilities, including: – Autism Spectrum Disorder/Condition – Asperger Syndrome	– Participants without a specific intellectual or developmental diagnosis – Studies referring only to ‘disabilities’ in general – Children and adolescents (under 18) – Families or carers
Focus/Topic	Participants' own experiences of love and romance, illustrated through verbatim quotations	– Studies on unrelated topics (e.g., friendship, sexuality without romantic/love context) – Studies focused on second‐hand accounts (e.g., from carers)
Methodology	– Qualitative data collection methods – Thematic analysis – Participant voice represented through direct quotations	– Quantitative methods only – No thematic analysis – No use of participant quotations
Date range	Published between 1998 and 2024	Published before 1998 or after 2024
Publication type	Peer‐reviewed journal articles	Non‐peer‐reviewed sources (e.g., books, dissertations, reports, grey literature)
Language	English	Any non‐English language
Geographical location	United Kingdom (UK)	Non‐UK‐based studies

### 3.1 Search Strategy

We devised different search term combinations, testing each for the number of relevant results. For example, the terms disab* and relationship* on their own were excluded due to the high volume of non‐relevant results they produced. We decided on the following search strings: (intellectual disab*, OR learning disab*, OR developmental disab*, OR retard*, OR PDD, OR pervasive developmental disorder*, OR learning difficult*) AND (lov*, OR intimate, OR romantic, OR dating, OR boyfriend*, OR girlfriend*, OR partner, OR marri*, OR sexual relationship*). The following eight databases identified 15,984 results: Academic Search Ultimate, ERIC, IBSS, PsycINFO, PubMed, Scopus, JSTOR, Web of Science. We also searched specialist intellectual and developmental disabilities journals and reference lists of included articles.

### 3.2 Identified Records

**FIGURE 1:**
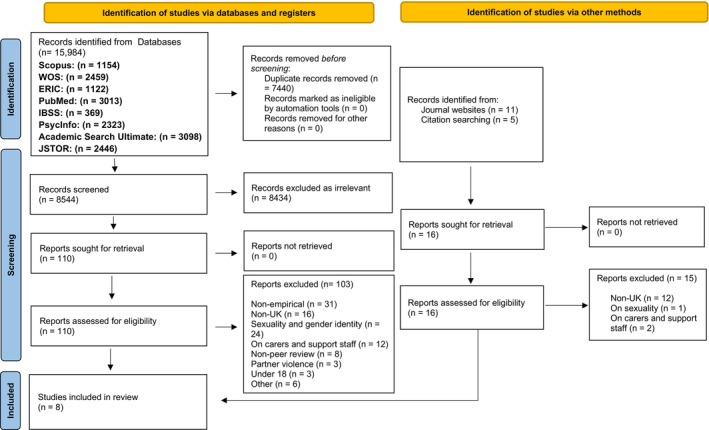
PRISMA flowchart of study selection (*Source:* Page et al. 2021).

After duplicates were excluded, the titles and abstracts of the remaining 8544 papers were screened for irrelevance. The resulting 126 papers were then screened in full text by all three authors with 118 excluded according to our review criteria and any discrepancies between authors were discussed until agreement was reached (see Figure 1). Although our inclusion criteria specified intellectual and developmental disabilities, the final eight papers for review concerned people with intellectual disabilities only.

*Step 3*: Reading the studies and quality appraisal.

Retrieved papers (*n* = 8) that ‘passed’ our full screening were re‐read several times by all three authors to immerse themselves in each paper's content and focus their thinking. All eight papers where subjected to a Critical Appraisal Skills Programme (CASP) Checklist: For Qualitative Research (CASP 2013) to assess each study for clarity of research aims, appropriateness of methodologies, data collection rigour, researcher reflexivity, ethical considerations, robustness of data analysis, and reporting essential systematic review components (Table 2). While some studies varied in reporting detail, overall the quality of the studies was strong. All of the studies scored positively to 7 or more of the 10 CASP questions with one study (Bates et al. 2016a scoring 10) and were therefore permitted to move to the next analytical step. This appraisal was conducted collaboratively by all three authors to ensure consistency and mitigate bias.

**TABLE 2:** CASP Analysis of included studies.

	White and Barnitt (2000)	Abbott and Burns (2007)	Lafferty et al. (2013)	Rushbrooke et al. (2014)	Bates et al. (2016b)	Bates et al. (2016a)	McCarthy et al. (2022)	Watchman et al. (2024)
1. Was there a clear statement of the aims of the research?	✓	0	✓	✓	✓	✓	✓	✓
2. Is a qualitative methodology appropriate?	✓	✓	✓	✓	✓	✓	✓	✓
3. Was the research design appropriate to address the aims of the research?	✓	✓	✓	✓	✓	✓	✓	✓
4. Was the recruitment strategy appropriate to the aims of the research?	0	✓	✓	✓	✓	✓	✓	✓
5. Was the data collected in a way that addressed the research issue?	✓	✓	✓	✓	✓	✓	✓	✓
6. Was the data collected in a way that addressed the research issue?	X	0	0	0	✓	✓	0	0
7. Have ethical issues been taken into consideration?	✓	✓	✓	✓	✓	✓	✓	✓
8. Was the data analysis sufficiently rigorous?	✓	✓	✓	✓	0	✓	✓	✓
9. Is there a clear statement of findings?	X	✓	✓	✓	✓	✓	✓	✓
10. How valuable is the research?	0	✓	✓	0	✓	✓	✓	✓
CASP Score	7	9	9.5	9	9.5	10	9.5	9.5

*Note:* ✓ = Yes, X = No.

*Step 4*: Determining how the studies were related.

We followed previous reviewers' applications of Noblit and Hare's meta‐ethnography (including Thomas and Harden 2008) by following three interlinked phases:

1. *Data Extraction*. Using a standardised template, the following study characteristics were extracted from each paper: Authors and publication year, sample size, age of participants, duration of study, methodological approach, participant involvement in the study, methods and analysis used, strengths, limitations, ethics and particularly whether consent had been gained from participants, impact and main findings (Table 3). Findings sections from each paper including participants' quotes and researcher interpretations were also repeatedly read line‐by‐line by the authors and extracted for relevance.
2. *The extracts were coded* with key relationships between the studies identified, providing a description of the original authors' findings that addressed our objectives;
3. *Emerging ideas for themes* were noted, with additional themes added.

**TABLE 3:** Characteristics of selected studies.

Author (year)	Sample size	Age (years)	Duration of study	Methodology	Participant involvement	Methods	Analysis	Strengths	Limitations	Ethics and consent	Impact and practicality	Results
White and Barnitt (2000)	8	18–35	Not specified	Qualitative, semi‐structured interviews	Direct participant interviews	Semi‐structured interviews	Hermeneutic phenomenology allowing for analysis of pre‐selected themes used in the interview questions	Highlights personal experiences	Small sample; all but one living in the same residential home. Ethnic diversity not stated	Consent obtained orally; ethical approval noted	Provides insight into intimate relationships of people with disabilities	All but one participant experienced satisfying relationships, mainly with others with intellectual disabilities. Overprotective parenting and care practices limited autonomy.
Abbott and Burns (2007)	20 (LGB)	22–59	2003–2004	Grounded theory & thematic analysis	Individual interviews about relationships, discrimination, & sexuality	One‐on‐one semi‐structured interviews lasting 1–3.5 h	Comparative & thematic coding	Unique focus on LGB individuals with intellectual disabilities. Rarely studied area	Staff self‐selection bias	Ethical approval obtained; multimedia aids for informed consent	Highlights systemic barriers faced by LGB individuals. Produces resources for advocacy & service improvement	Barriers to forming relationships included stigma, social isolation, & carer negativity. Participants desired intimacy despite discrimination.
Lafferty et al. (2013)	16 (8 couples)	26–65	Not specified	Grounded theory	Joint & individual interviews	Dyadic & one‐to‐one interviews	Open coding, constant comparative method	Emphasizes relationship dynamics	Gatekeeper‐selected sample. Ethnic diversity not stated	Accessible consent obtained; ethical approval granted	Highlights value of close relationships and recommends support strategies	Long‐term heterosexual relationships emphasized mutual care, commitment, & external controls by caregivers.
Rushbrooke et al. (2014)	9	21–58	Not specified	Interpretative phenomenological Analysis (IPA)	Direct participant interviews	Semi‐structured interviews	IPA with double hermeneutic approach	Rich qualitative insights	Ethnic diversity not stated. Limited generalizability	Informed consent: ethical approval noted	Supports caregiver understanding & policy development	Explored stigma, sexual identity, & relational autonomy. Control over relationships is often imposed by others.
Bates et al. (2016b)	11	< 35–60+	Not specified	Hermeneutic phenomenology (Van Manen)	Interviews about the importance of romantic love for people with disabilities	In‐depth, semi‐structured interviews	Thematic analysis (Van Manen's method)	Explores a marginalized topic. Accessible design. Insight into staff roles	Small sample size; excludes homosexual relationships. Biased recruitment	Ethical approval granted; ongoing consent checks	Revised Maslow's hierarchy to emphasize the importance of love for individuals with learning disabilities	Explored societal & care constraints on relational possibilities. Highlighted the importance of affection and companionship.
Bates et al. (2016a)	11	< 35–60+	Not specified	Hermeneutic phenomenology (Van Manen)	Interviews focusing on partner selection traits valued by participants	In‐depth, semi‐structured interviews	Thematic analysis (Van Manen's method)	Novel exploration of partner selection. Rich narratives	Small sample size. Focus on heterosexual relationships; ethnic diversity not stated	Ethical approval granted; consent ensured	Highlights support gaps for relationships. Advocates for inclusive services	Relationships often formed in segregated environments. Criteria for partner selection included kindness, companionship, & commitment.
McCarthy et al. (2022)	40	22–71	October 2017–April 2019	Semi‐structured in‐depth interviews	Participants shaped the study; advisory groups engaged	Semi‐structured interviews	Thematic analysis using Braun & Clarke framework	Inclusion of diverse voices & perspectives	Majority heterosexual participants	Ethical approval obtained; easy‐read materials provided	Emphasized importance of loving relationships for well‐being	Diverse sample included 25% *BAME* individuals. Stigma and caregiver attitudes impacted relational autonomy.
Watchman et al. (2024)	8 couples plus input from 13 staff & 4 family members	43–91	8 months in 2022	Narrative life‐story & multiple case study approach	Couples & advisory groups co‐developed tools and analyses	Interviews (individual and couple), visual supports, and life‐story mapping	Deductive and inductive coding; thematic analysis with NVivo	Focus on overlooked population; innovative visual methods	Small sample size, UK‐based, all White participants	Ethical approval obtained; careful support to minimize distress	Highlighted needs of aging couples with intellectual disability & dementia	Explored resilience in long‐term relationships facing dementia. Emphasized emotional bonds despite health adversities.

*Step 5*: Translating the studies into one another.

Key themes were identified to build new ‘lines of argument’ (Noblit and Hare 1988, 38).

*Step 6*: Synthesising translations.

Whilst previous meta‐ethnographies have tended to synthesise only the authors' interpretations of data (Atkins et al. 2008) or their themes (Noblit and Hare 1988, 38–47), our small number of studies meant that we could synthesise all data presented in each paper's findings sections including quotations. This allowed our data to consist of participants' own experiences, allowing their ‘voices’ to be heard.

*Step 7*: Expressing the synthesis.

The following section reports the output of the synthesis.

## 4 Results

### 4.1 Characteristics of Studies

All eight studies used phenomenology, grounded theory, and/or participatory methods to delve into individuals' nuanced experiences of love and therefore sample sizes were small ranging from 8 to 40 participants (total *n* = 123 across all studies). These methodologies align with our chosen social‐affirmation framework since they focus on participants' subjective views and experiences. White and Barnitt (2000), Rushbrooke et al. (2014) and Bates et al. (2016a, 2016b) adopted variants of hermeneutic phenomenology. These are particularly well‐suited to examining the lived experiences of people with intellectual disabilities when the concepts being explored are rather abstract, including love, relationships, and personal agency. The use of phenomenology points to participant‐centred designs. Semi‐structured interviews and focus groups, allow flexibility to adapt to participants' needs and preferences for communication. Abbott and Burns (2007) and Lafferty et al. (2013) both used grounded theory. This method allows themes to emerge on their own rather than being specified a priori. This recursive process is particularly helpful when working with subtle experiences, as happens in intimate relationships or acts of self‐advocacy. Grounded theory also enables different perspectives to emerge, which aides making sense of relationship dynamics and support networks. Participatory methods used by (McCarthy et al. 2022) and (Watchman et al. 2024) allowed participants to have agency in the research process including research design, data gathering, and interpretation. The age range of our participants was broad (from 18 years old to 91 years old). This highlights how the ability to think about love and to love others does not disappear along ageing for people with intellectual disabilities, corroborating Määttä's (2011) finding that love was highly important for 117 people without intellectual disabilities who were aged between 50 and 91.

Adapted communication tools, including simplified, unambiguous language (Bates et al. 2016a, 2016b), accessible materials/information sheets (McCarthy et al. 2022; Watchman et al. 2024), and visual aids (Watchman et al. 2024; Abbott and Burns 2007) were particularly beneficial for individuals with verbal communication difficulties, enabling their perspectives to be meaningfully included. However, we noted that participants with higher‐level support needs (e.g., people who might be described as ‘non‐verbal’, or those with challenging behaviours) were generally absent from the studies reviewed. All the studies had considered ethical issues and sought to mitigate them.

### 4.2 Key Themes About Love

Using thematic synthesis we identified three overlapping key themes: how people with intellectual disabilities conceptualised love; how they experienced love; and barriers to finding and sustaining love. All three themes are sensitive to differences in age, gender, sexual orientation, living arrangements, and relational context. The themes fit the social‐affirmation model by demonstrating how society sets individuals apart by impeding their ideas of love and goals to love and be loved.

#### 4.2.1 Theme One: Conceptualisation of Love

As discussed above, the pursuit and maintenance of love as an important life domain has generally been ignored for people with intellectual disabilities. This is a result of stigmatised views that people with intellectual disabilities are unable to or shouldn't have romantic relationships. Rather they have been viewed as intellectually unable and/or needing protection from intimacy. We wanted to challenge these assumptions by exploring how love is understood by participants; how they describe love, and what it means to them in their own words. Some participants had communication difficulties, but all were able to demonstrate an ability to express how they understood and defined love. This was encapsulated into three subthemes: emotional fulfilment and companionship, mutual support, and resilient love.

##### 4.2.1.1 Subtheme: Emotional Fulfilment and Companionship

Love was described as a key driver of happiness, stability and emotional fulfilment. Participants in McCarthy et al. (2022, 958–959) study described love in the following ways:

Love brings you happiness, brings you contentment.

when I say I love my girlfriend, I love her like she's my universe, she's everything…But like, I always say to her, ‘I love you until infinity and beyond, no return. She can't beat that; you can't beat infinity.

Companionship characterised by kindness and fun also formed participant's definitions of love (Bates et al. 2016a, 607):

Joe: She's a great person in my life, friendly, kind, funny and fun to be with [turning to Carrie]. I love you… (Bates et al. 2016a, 607)

as did affection and appreciation:

Trisha: It was that he loved me, and he showed me how much he really liked me (White and Barnitt 2000, 273).

I like buying things… taking them out for a meal…surprising them or buying them flowers and stuff (McCarthy et al. 2022, 959).

Pearl, Sarah and Joe articulated their love for others as all‐encompassing:

[Partner] means a lot to me…Er, I couldn't part with him. Right, I love him that much, it's part of in me heart (Pearl) (Rushbrooke et al. 2014, 534).

It is the best thing that ever happened to me. I wouldn't be without him (Sarah) (Lafferty et al. 2013, 1082).

So happy about the person I am married to, and also, she is my soul mate (Joe) (Bates et al. 2016b, 68).

Finally, the following participant articulated how they ‘fell in love’ by describing an intellectual and emotional awakening or ‘ah ha’ moment:

It was just that spark and seeing her for the first time, and something just clicked in me (Abbott and Burns 2007, 32).

These accounts iterate how love was not conceptualised as merely a romantic ideal. Rather, participants painted an image of love as a deep emotional experience that encapsulated companionship, acceptance, and fulfilment.

##### 4.2.1.2 Subtheme: Physical and Emotional Intimacy

Physicality, especially ‘a significant amount of affection such as kissing and cuddling’ and holding hands (Bates et al. 2016, b, 68) was a fundamental aspect of participants' conceptualisation of love. This form of intimacy represented emotional closeness, and feelings of security, and being wanted. One participant from Rushbrooke et al. (2014, 536) study described their sexual relationship as:

Very romantic…Sometimes I ask him to massage me…And it makes me relaxed, and then, one thing then leads to another (Joanne)

In a similar vein, a gradual approach to intimacy was suggested by another participant:

I think we need to get to know each other, what each other likes, respect each other. And I believe that if you just go for sex straightaway, if it's not what each other expects, it can just blow the whole thing out of the water, really (Abbott and Burns 2007, 31).

The above quote challenges societal narratives of sexual milestones by positing how intimacy can grow through trust, patience, and mutual respect and how it is highly individualised. Nonsexual forms of warmth and delight were also regarded as equally important demonstrations of love for many:

I used to walk round the street holding hands (Adam) (Rushbrooke et al. 2014, 536).

[When] we're together, we talk about things ourselves and that's just lovely… (Anne) (Lafferty et al. 2013, 1080)

We enjoy going to shows at the opera house…we go on walks together…we go out for meals and that, but mainly just being together (Sarah) (Lafferty et al. 2013, 1080)

##### 4.2.1.3 Subtheme: Mutual Support

Participants' definition of love also included the idea of reciprocity, defined by exchanges of emotional and practical support whereby each partner addressed the needs and strengths of the other:

Well, I want someone to love and care for me, and I want to care for them as well. It works both ways. You can't give all the loving. They've got to give it back, haven't they? (Pauline) (Abbott and Burns 2007, 32).

One couple described their synergistic love in terms of interdependency:

[We are] more independent, [although] we depend on each other, I depend on her and she depends on me… [we are] more independent and more going…. forwards instead of backwards (Michael) (Lafferty et al. 2013, 1080).

These accounts speak to relationships characterised by a sense of partnership, bringing stability and mutual affirmation, one participant (Sarah) explaining what she loved about her partner:

Everything and everything—her body! I don't know, she protects me, she supports me, she listens to me, helps me out when I need help. She is with me 24‐7 (Abbott and Burns 2007, 32).

What seemed to be missing from participant's narratives was the idea that love could be unrequited or painful when reciprocity was missing. Interviews may have omitted questions about ‘loving from a distance’, one‐sided love, and the frustrations that go with these aspects of relationships.

##### 4.2.1.4 Subtheme: Love Is…Commitment and Devotion

Commitment and devotion were facets of love that gave participants a sense of emotional security and direction, as well as happiness:

I've been alone all my life…and I would live with him forever then (Daffyd) (Abbott and Burns 2007, 32).

Love was therefore conceptualised as a deeply personal bond (Watchman et al. 2024, 5) arguing:

The eight couples in our study had been together for a combined total of 170 years, highlighting longevity in relationships, with most lasting for more than 20 years.

Participants also understood love as transcending relational difficulties with steadfast commitment, one participant revealing:

We have arguments, but we would never be apart from each other…we have our wee ups and downs, but I wouldn't change him for the world, I love him (Sarah) (Lafferty et al. 2013, 1082).

Although observed rather than generated from interviews, (Watchman et al. 2024) evidenced resilient loving bonds between couples in the face of external circumstances. Whilst illness or declining health altered the dynamics of their everyday lives, the core commitment and emotional connection between partners endured and defined love, Watchman et al. (2024, 6) stating:

As dementia progressed, interactions between partners were described as decreasing, with more conflict for some. However, despite changes to relationships there continued to be love and care between partners.

Similarly, Anne in (Lafferty et al. 2013) study clearly defined love in terms of ongoing support:

Now, I support Brian through thick and thin, that's what love is (Anne) (Lafferty et al. 2013, 1080).

These findings challenge stereotypical attitudes of people with intellectual disabilities being dependent on others. Instead, they portray relational dynamics that define love and are marked by sustained mutual support and adaptive caregiving.

#### 4.2.2 Theme Two: Experiences of Being in Love and Living Without Love

All eight studies discussed participants' lived experiences regarding love and how it materialised in their everyday lives. Love as an antidote to loneliness was also discussed.

##### 4.2.2.1 Subtheme: The Experience of ‘Being in Love’

For many participants, love was experienced as a therapeutic, restorative and resilient agent, mediating healing from past traumas and buffering life's adversities (Abbott and Burns 2007; Lafferty et al. 2013; McCarthy et al. 2022; Watchman et al. 2024). Bates et al. (2016b) described the healing potential of intimate relationships, whereby love counterbalanced previous harms and enabled emotional recovery:

The love they felt for a partner appeared reparative in regard to abuse some participants had previously experienced (p. 67).

Affection also engendered resilience by affirming participants' self‐worth, enabling them to navigate systemic marginalisation, as exemplified by McCarthy et al. (2022, 959) participants:

I would say if others have the opportunity to achieve what we have, I would say grab it with both hands and don't waste time.

I say, love is a wonderful thing.

This speaks to the empowerment that love brings, providing permission and inspiration to seize possibilities of connection. The experience of persistent love, even in the face of the ravages of Alzheimer's, was described nostalgically by one partner without dementia in (Watchman et al. 2024) study:

We used to have a nice time together, even those times, like she used to hold my hand, I used to hold her hand and we used to cuddle and kiss, and she'd go ‘sorry’, I'd say, ‘Oh don't worry about it, it's all right, it wasn't your fault’. And then we'd have a cuddle and then we'd be all right together (couple 2) (p. 6).

Support and solidarity in relationships also served to shore up resilience in the face of external challenges, including prejudice and discrimination, as well as everyday relationship issues as described by Abbott and Burns (2007, 31) concerning a lesbian couple:

They spoke openly about how much they loved and supported each other and the difficulties they faced with their families and day‐to‐day tensions and arguments in the relationship.

Thus, shared experiences of love and mutual care enabled participants to rise above their interpersonal and familial challenges together. For others, relationships were experienced as cultivating personal growth and independence:

When you get married you move away from your family, and you make your own life. If you make mistakes, well you make them, but you have only yourself to blame, you don't want to start blaming somebody else (Sarah) (Lafferty et al. 2013, 1081).

Here, love elicited maturity, autonomy, accountability and responsibility regarding decision‐making.

##### 4.2.2.2 Subtheme: Love as an Antidote to Loneliness

Loneliness and the desire for partnerships, intimacy and commitment were significant subthemes in all the reviewed papers. Studies demonstrated how loneliness was understood by participants as an outcome of an unanswered quest for love, or a love lost. Though reflecting on their desire to connect, participants shed light not only on their struggles to love and be loved, but also on their resilience and capacity to continue seeking meaningful relationships. Loneliness was poignantly voiced through participants' narratives; the lack of romantic or intimate relationships, exacerbated by social marginalisation, added to feelings of loneliness, as exemplified below:

Sometimes I get lonely, and I think if I had got somebody who I could trust it would make me more happier (McCarthy et al. 2022, 959).

When you've a learning disability, it can be very lonely. You feel as if the whole world is coming down on top of you (Anne) (Lafferty et al. 2013, 1080).

The latter statement describes the emotional burden of loneliness on participants, placing it into the broader context of public neglect and the lack of opportunities for persons with intellectual disabilities to be involved in social or romantic interactions. Similarly, another participant lamented:

Emma: Well, the thing that makes me happy being with someone is, I would rather live with someone, a partner, than being on my own because I don't like being on my own (Bates et al. 2016b, 68).

Participants echoed the universal desire for connection and belonging which was further intensified by their struggles with prejudice; (Abbott and Burns 2007) recounting Jim's story of losing his partner to illustrate the layered complexity of loneliness and grief:

…The two men had kept the relationship a secret from staff in the home where they lived. Sadly, this meant that when Jim's friend died, nobody took much care in telling him. He kept his grief a secret for several years: Jim: ‘The supervisor just said he had a heart attack, just out of the blue like that. So, em, [long pause] so like, em, I felt, [pause] quite lonely, so like, em, it's sort of like suddenly it's over because, [pause] bit of a shame’ (Abbott and Burns 2007, 31).

The secrecy of Jim's relationship reflects the stigmatisation of people with intellectual disabilities regarding romantic and sexual relationships, particularly within care settings. In Jim's case, he risked societal intersectional stigma of being disabled and in a same‐sex relationship, thereby compelling him to hide his relationship due to fear of judgement and restrictions imposed by paid carers. (Rushbrooke et al. 2014, 535) also noted, ‘Participants who had (or sought) same‐sex partners spoke of hiding their sexuality from others’. The stigmatisation Jim experienced that obstructed his ability to grieve openly for his lost love overlaps with theme three below concerning barriers society constructs.

#### 4.2.3 Theme Three: Barriers to Finding and Sustaining Loving Relationships

Romantic love was a significant aspiration for all participants. However, systemic and interpersonal barriers, including caregiver control, limited social opportunities, discrimination and lack of privacy often frustrated this goal.

##### 4.2.3.1 Subtheme: Systemic and Caregiver Control

Residential homes with structural constraints and social milieus characterised by social control stifled participants from freely engaging in emotional and physical intimacy. These environments also hindered the development of romantic relationships. One participant's frustration was summed up thus: ‘We just wanna cuddle and we can't’ (Bates et al. 2016b, 68). Restrictions to meeting, establishing and maintaining loving relationships were not only confined to residential homes. Care policies and practices, systemic attitudes of paid carers, and power dynamics reflected broader societal biases about people with intellectual disabilities and their capacity for love and intimacy. Despite these obstacles, participants continued to pursue intimacy as a means of connecting and expressing themselves. The necessity of carers for logistical aspects of relationships was also acknowledged, (Bates et al. 2016b, 68) stating:

Mary and Peter [study participants] claimed that staff were responsible for organising dates (outside the home). It appeared that without staff support, participants with higher needs would have not been able to establish a relationship.

The role caregivers played revealed a double‐edged sword. Their ‘power’ either facilitated or restricted romantic pursuits, depending on their attitudes and service policies. Participants' overall frustration with their lack of romantic autonomy was summed up affectingly by Georgia:

People think that they can rule you because you've got a disability… ‘You can't choose the colour that you like. As well as your boyfriend you can't pick’. I say, ‘Hang on there you! Who do you think you're talking to?’ Doesn't mean that I get me support from support staff. You can choose who I love and who I like, and you pick the man for me (Georgia) (Rushbrooke et al. 2014, 537).

Carer priorities and attitudes also emerged as barriers for romantic exploration, for example:

Most staff said that they did not routinely engage in client issues having to do with relationships and sexuality whether clients were heterosexual, homosexual, or bisexual [and] …the issue was generally ignored until there was a crisis….the local day centre found a couple having sex in the shed – and guess what the day centre did to deal with the problem? They took down the shed. I think that says it all, doesn't it? (Abbott and Burns 2007, 34).

Similar inhibitive attitudes of family members were also found, exemplified by this response to parental involvement in a participant's relationship:

Well actually the only ones were our parents and then I stepped in and said, “Now look I know that we can make the decisions, and I know you like to give advice, but we are the ones to make the decisions” (Dave) (White and Barnitt 2000, 274)

Despite these obstacles, participants continued to pursue intimacy as a means of connecting and expressing themselves.

##### 4.2.3.2 Subtheme: Lack of Privacy

Participants complained about systemic violations of their privacy, noting how restrictive care practices undermined their relationships. For instance, some caregivers withheld personal correspondence:

I have letters from him [boyfriend] sometimes and the staff keep them in the office in a box … a box for personal things and letters…(Sheila). And how does it make you feel? (Interviewer) I don't mind but it gets on my nerves a bit (Sheila) (White and Barnitt 2000, 274).

Spaces for love and intimacy to flourish were often policed rather than supported:

People who lived in a ‘group environment’ experienced a lack of privacy, with locks not always provided on bedroom doors and staff not knocking prior to entry (Bates et al. 2016a, 603).

Such intrusions disregarded bodily privacy, reinforcing feelings of disempowerment and extended beyond group homes to semi‐independent living arrangements:

She [manager of a residential learning disability service that Sean lived above in an independent flat] told me last week that I'm not allowed to bring a man back—to have sex… ‘cause [she said] it's embarrassing’ (Sean) (Abbott and Burns 2007, 33).

I just don't get no privacy with my boyfriend…staff make sure I'm okay and make sure we're fine and stuff like that (McCarthy et al. 2022, 961).

Having support there all the time we couldn't feel like we could do things what normal people would do in a relationship. ‘Like kiss, cuddle, hold hands’ (Rushbrooke et al. 2014, 538).

These cases underline the pervasiveness of carer control, whereby even those living semi‐independently were still subjected to surveillance, moral judgements and restrictions on their personal choices, hindering spontaneity or freedom to build these important, personal connections and curtailing opportunities for romantic exploration.

##### 4.2.3.3 Subtheme: Limited Social Opportunities for Romance

Economic constraints were significant in limiting participants' opportunities for romance:

It costs £12 for a cinema ticket in town; I can't afford that (McCarthy et al. 2022, 960).

Dense social networks that rarely involved people other than people with intellectual disabilities also curbed opportunities to meet a romantic partner, cementing a cycle of insularity and romantic isolation:

I've been trying hard to find one, but it's looking for a needle in a haystack (Jeremy) (Rushbrooke et al. 2014, 536).

Participants also referred to the lack of proactive carer interventions which left them feeling unsupported and unable to overcome their apprehensions in social settings, one participant arguing:

Staff should have done more. Like maybe assist you more to find someone, to go out there and help you (McCarthy et al. 2022, 961).

While positioned to facilitate social opportunities, passive or restrictive carer attitudes further entrenched participants' isolation. The emotional outcomes of this isolation were profound, with some participants reporting emotional distress as exemplified by Ann in Abbott and Burns's (2007) study:

I am isolated, and I am lonely… It makes me so angry and so frustrated. Why am I getting punished? Why am I having the life of a child instead of an adult? (p. 35)

Ann's anguish reflects an intersection of isolation, societal infantilisation and unmet relational needs; her plea for recognition as an autonomous adult highlights the systemic failure to address the emotional well‐being of people with intellectual disabilities. For some, the pain of isolation extended to mental health consequences, including thoughts of self‐harm and suicide:

Two men spoke to us of having tried to commit suicide, and one woman had harmed herself and thought about suicide (Abbott and Burns 2007, 35).

Pervasive barriers to romantic connection including economic constraints, forced segregation, and lack of care support therefore intersected, reinforcing isolation and discouraging the development and maintenance of loving relationships.

##### 4.2.3.4 Subtheme: Discrimination

A general lack of understanding of the importance of love as a wellbeing domain for people with intellectual disabilities was underpinned by stigmatised attitudes. These mindsets that encapsulated infantilisation led to discriminatory practice that reduced opportunities for connections, leading to the marginalisation and disempowerment of individuals. For some participants, conversations between carers about their own relationships that gave the impression of commonplace exeriences of felt exclusionary:

I felt so excluded because I wasn't having the babies, I wasn't living with anyone, in a relationship with them. I was off on my own. I felt so excluded (McCarthy et al. 2022, 959)

Yet desires for intimacy expressed by people with intellectual disabilities were sometimes met with derision, Debbie being teased by carers about her relationship:

Me support workers always say, ‘Oooh, are you gonna dance with him? Are you gonna kiss him? Trying to embarrass me in front of him’. So yeah, I get a bit embarrassed (Debbie) (Rushbrooke et al. 2014, 538).

Such actions served to discredit participants' dignity, creating a culture of humiliation rather than encouragement. Moreover, although participants wanted to own their decisions including mistakes about relationships, they were often bound by gatekeeping practices by their families as well as carers:

I know my family care, but they shouldn't stop me, because I'm an adult. Like, ‘I'm human, let me make my own mistakes and then I know, okay, that was a mistake’…But I don't know if it's going to be a mistake if I don't try… (McCarthy et al. 2022, 961).

Curtailing the opportunity to make mistakes served to hinder personal growth, robbing individuals of the core human process of learning through trial and error. The stigmatisation of non‐heteronormative relationships added another layer of complexity for participants, (Abbott and Burns 2007, 35) stating:

Staff attitudes toward clients' relationships could determine completely the way in which these relationships are perceived by others…Same‐sex relationships are automatically viewed as problematic.

This discriminatory stance reflects the intersectionality of stigma, whereby societal biases against both disability and (in this case) LGBTQ identities conjoin to narrow relational opportunities and acceptance. The included studies unfortunately revealed deeply ingrained prejudice that undermined relational agency of participants.

## 5 Discussion

To our knowledge, this systematic review is the first to synthesise evidence concerning both experiences of love as well as how love is conceptualised by people with intellectual disabilities. Participants' narratives showed an ability to articulate their understanding of love clearly using examples of their own relationships. This challenges the conceived wisdom that people with intellectual disabilities cannot understand what love is or means to them.

The dearth of research in the UK investigating this topic (we only found eight studies) and the lack of knowledge translation of research findings to practitioners (Dew and Boydell 2017) may explain why care services continue to view love as insignificant compared to other life domains such as employment (Forrester‐Jones et al. 2021). European research appears to have given more attention to the topic. (Mattila et al. 2016) interviewed seven Finnish young adults with mild intellectual disabilities finding that they could describe love in terms of emotions and concrete acts but not in relation to knowledge and skills. Using a theoretical model to investigate variables that described and explained love for 376 people with intellectual disabilities in Spain, (Arias et al. 2009) found that commitment, stability, passion and physiological excitement, intimacy and romanticism were all ‘love’ constructs. They also found that perceptions of love were largely idealised and affected by the context including family interference. Similarly, in our review emotional fulfilment, companionship, affection, mutual care and resilience were fundamental tenets of love and drivers to happiness, stability and a sense of belonging. Experiences of love encapsulated a desire for physical and emotional intimacy (although not necessarily sexual) as an antidote to loneliness, as well as commitment and devotion.

These truths jar with societal attitudes and expectations of people with intellectual disabilities as dependents, unable to provide or reciprocate love. Rather, through the lens of the social‐affirmation model (Swain and French 2000; Malli and Forrester‐Jones 2025) relationships cited in the review portrayed sophisticated expressions of love that moved beyond the limitations society placed on them. The many long‐term and stable relationships depicted in our review are a testament to participants' commitment to their relationships in the face of structural and social obstacles they experienced.

Despite these clear exhibitions of love, policy and practice restrictions limited opportunities for romance. House rules, often justified under the guise of safety or propriety, disregarded the emotional and relational needs of residents much like infringements of their spiritual needs (Forrester‐Jones and Raji 2025). Systemic violations of privacy and autonomy reflect a broader cultural refusal—based on the biological/medical deficit model of disability—to recognise the validity of relational agency of people with intellectual disabilities (Parchomiuk 2013).

(O'Brolcháin and Gordijn 2019) warn of increased privacy threats with the advancement of smart homes for people with dementia and those with intellectual disabilities (dementia disproportionately affects people with intellectual disabilities [Jacobs et al. 2023, 241]). Smart homes promise huge benefits. Visual, tactile and signalling devises as well as speech recognition (Alam et al. 2012) and personal ‘wearables’ including body temperature and oxygen saturation sensors; all offer opportunities for greater individual autonomy, security and safety (Majumder et al. 2017). However, their use raises ethical issues including privacy, with individuals being at risk of having their movements monitored by care staff or family members. Fear of being ‘watched’ remotely could further curtail intimate moments between partners. None of the participants in our review lived in smart homes but we argue that the social‐affirmation model of disability needs to underpin technological advancements to offset a return to medical model alignment.

Our systematic review encompassed studies conducted between 2000 and 2024. We therefore anticipated that this 24‐year period would reveal changes or trends in the literature, particularly regarding evolving perspectives on love and relationships for individuals with intellectual disabilities allied with social models of support. All the studies used a phenomenological approach that sought to understand love from the perspective of people's own unique experiences. Yet despite the rapid development of alternative and augmentative communication styles over the last two decades to authentically gather the views of people with intellectual disabilities (e.g., Cambridge and Forrester‐Jones 2003) it was not until 2024 that such supports were included (see Watchman et al. 2024).

It was disheartening to discover that stigma and discrimination continues to limit individuals with intellectual disabilities in establishing and sustaining romantic relationships. Nevertheless, we also observed encouraging trends in the literature, such as carers acknowledging the genuineness of loving relationships between individuals, alongside recognition of their desire and need for love. Furthermore, as the review period progressed, studies increasingly included diverse populations of people with intellectual disabilities indicating a more inclusive approach.

### 5.1 Limitations of Studies

Six of the eight studies revealed limitations in methodological quality, particularly around data collection, which meant that the research issue was not always fully addressed. Analytical depths and some researcher reflexivity gaps were also identified, suggesting further refinement for future studies. The absence of participants with higher support needs from almost all the studies reflects the continuous inadvertent exclusion of this group of people. This is often due to limited funding to develop appropriate inclusive strategies, recruitment that does not involve contacting potential participants directly (Cleaver et al. 2010) or lack of expertise to support alternative and augmentative communication styles (Cambridge and Forrester‐Jones 2003). Given the ethical imperative of research that is more inclusive of disability spectrums we would hope that this situation will change.

### 5.2 Limitations of Our Systematic Review

Our review was tied to a larger UK‐based empirical study investigating specialist dating agencies for people with intellectual disabilities (McCarthy et al. 2020) and therefore limited to UK studies. A more global review would arguably provide further insights on the topic as well as examples of good practice.

## 6 Conclusion

Through systematically reviewing eight UK‐based studies over a 24‐year period we traced three themes: conceptualisation of love; experiences of being in love and living without love; and barriers to finding and sustaining loving relationships. Despite cognitive and communication difficulties, all participants (*n* = 123 across the eight papers) were able to demonstrate an ability to express how they understood and defined love. Their definitions included emotional fulfilment, companionship, kindness, mutual support, and resilience. In terms of how love was experienced, participants reported it as a therapeutic, restorative and resilient agent, mediating healing from past traumas and buffering life's adversities. It also mediated feelings of empowerment and self‐worth—enabling people to view romantic opportunities as more reachable, fostering confidence to ‘seize the day’. Love was also an antidote to loneliness in what is ostensibly a marginalising world. Although all participants aspired to ‘be in love’, systemic and interpersonal barriers, including caregiver control, limited social opportunities, discriminating caregiver behaviours and lack of privacy often thwarted this goal.

Given the legal right to a private life enshrined in international law and ratified in UK law and policy, there is an obvious need for a cultural shift in thinking and practice around romantic relationships for people with intellectual disabilities (Correa et al. 2022). Training on relational care support that promotes empathy and respect for love would be a start. Care plans should specifically acknowledge and respond to individuals' capacity to feel and express love and residential and other care environments should affirm emotional and relational needs thereby dismantling ‘institutional walls’ (Rushbrooke et al. 2014) that constrain love.

Focusing on UK studies revealed (a) a dearth of research on the topic of love, and (b) a lack of service application for enabling people with intellectual disabilities to experience loving relationships as a fundamental right. We argue for a more global review to uncover good practice in this important area.

## Conflicts of Interest

The authors declare no conflicts of interest.

## Data Availability

Data sharing is not applicable to this article as no new data were created or analyzed in this study.

## Endnotes

^1^
The term learning disabilities is commonly used in the UK to mean intellectual disabilities. We refer to intellectual disabilities when quoting authors who use the term learning disabilities.

^2^
Lesbian, gay, bisexual, transgender, queer and/or questioning. Since these publications, the term has expanded to also include intersex, asexual or agender, and two‐spirit (i.e., LGBTQIA2S+).

## References

[jar70132-bib-0001] Abbott, D. , and J. Burns . 2007. “What's Love Got to Do With It? Experiences of Lesbian, Gay, and Bisexual People With Intellectual Disabilities in the United Kingdom and Views of the Staff Who Support Them.” Sexuality Research & Social Policy 4, no. 1: 27–39. 10.1525/srsp.2007.4.1.27.

[jar70132-bib-0002] * Abbott, D. 2015. “Love in a Cold Climate: Changes in the Fortunes of LGBT Men and Women With Learning Disabilities.” British Journal of Learning Disabilities 43, no. 2: 100–105. 10.1111/bld.12131.

[jar70132-bib-0070] Alam, M. R. , M. B. I. Reaz , and M. A. M. Ali . 2012. “A Review of Smart Homes—Past, Present, and Future.” IEEE Journals & Magazine. IEEE Transactions on Systems Man and Cybernetics Part C (Applications and Reviews).

[jar70132-bib-0003] Amado, A. R. N. 1993. Friendships and Community Connections Between People With and Without Developmental Disabilities (Foreword by C. Wieck). Paul H. Brookes.

[jar70132-bib-0004] Arias, B. , A. Overjero , and R. Morentin . 2009. “Love and Emotional Well‐Being in People With Intellectual Disabilities.” Spanish Journal of Psychology 12, no. 1: 204–216. 10.1017/S113874160000161X. 19476233

[jar70132-bib-0005] Atkins, S. , S. Lewin , H. Smith , M. Engel , A. Fretheim , and J. Volmink . 2008. “Conducting a Meta‐Ethnography of Qualitative Literature: Lessons Learnt.” BMC Medical Research Methodology 8: 21. 10.1186/1471-2288-8-21. 18416812 PMC2374791

[jar70132-bib-0006] Azzopardi Lane, C. L. , P. Cambridge , and G. Murphy . 2019. “Muted Voices: The Unexplored Sexuality of Young Persons With Learning Disability in Malta.” British Journal of Learning Disabilities 47, no. 3: 156–164. 10.1111/bld.12266.

[jar70132-bib-0007] Baines, S. , E. Emerson , J. Robertson , and C. Hatton . 2018. “Sexual Activity and Sexual Health Among Young Adults With and Without Mild/Moderate Intellectual Disability.” BMC Public Health 18: 667. 10.1186/s12889-018-5572-9. 29843657 PMC5975712

[jar70132-bib-0011] Bates, C. 2019. “Supported Loving – Developing a National Network to Support Positive Intimate Relationships for People With Learning Disabilities.” Tizard Learning Disability Review 24, no. 1: 13–19. 10.1108/TLDR-06-2018-0017.

[jar70132-bib-0008] Bates, C. 2020. ““It's Nothing to Be Ashamed of, I'm Like, I'm Bisexual and I Love Women, I Like Men”: Being a Bisexual Person With an Intellectual Disability.” Journal of Bisexuality 20, no. 4: 493–513. 10.1080/15299716.2020.1836544.

[jar70132-bib-0009] Bates, C. , M. McCarthy , K. Milne‐Skillman , N. Elson , R. Forrester‐Jones , and S. Hunt . 2020. ““Always Trying to Walk a Bit of a Tightrope”: The Role of Social Care Staff in Supporting Adults With Intellectual and Developmental Disabilities to Develop and Maintain Loving Relationships.” British Journal of Learning Disabilities 48, no. 4: 261–268. 10.1111/bld.12320.

[jar70132-bib-0010] Bates, C. , M. McCarthy , K. Milne‐Skillman , N. Elson , S. Hunt , and R. Forrester‐Jones . 2021. ““She Misses the Subtleties and I Have to Help – Help to Make the Invisible Visible”: Parents' Role in Supporting Adults With Intellectual and Developmental Disabilities With Intimate Relationships.” International Journal of Care and Caring 5, no. 3: 489–507. 10.1332/239788220X16081401542782.

[jar70132-bib-0012] * Bates, C. , L. Terry , and K. Popple . 2016a. “Partner Selection for People With Intellectual Disabilities.” Journal of Applied Research in Intellectual Disabilities 29, no. 2: 602–611. 10.1111/jar.12254. 26996512

[jar70132-bib-0013] * Bates, C. , L. Terry , and K. Popple . 2016b. “The Importance of Romantic Love to People With Learning Disabilities.” British Journal of Learning Disabilities 45, no. 1: 64–72. 10.1111/bld.12177.

[jar70132-bib-0014] Black, R. S. , and R. R. Kammes . 2019. “Restrictions, Power, Companionship, and Intimacy: A Metasynthesis of People With Intellectual Disability Speaking About Sex and Relationships.” Intellectual and Developmental Disabilities 57, no. 3: 212–233. 10.1352/1934-9556-57.3.212. 31120408

[jar70132-bib-0015] Brown, M. , and E. McCann . 2018. “Sexuality Issues and the Voices of Adults With Intellectual Disabilities: A Systematic Review of the Literature.” Research in Developmental Disabilities 74: 124–138. 10.1016/j.ridd.2018.01.009. 29413427

[jar70132-bib-0016] Bunyan, S. , N. Clark , A. Herranz , et al. 1986. “Mental Handicap: Human Rights and Relationships.” Professional Nurse 2, no. 2: 41–43. 3642581

[jar70132-bib-0018] Cambridge, P. 1997. HIV, Sex and Learning Disability: A Staff Training and Sex Education Resource for Working on HIV and With Men With Learning Disabilities Who Have Sex With Men. Pavilion.

[jar70132-bib-0017] Cambridge, P. , and R. Forrester‐Jones . 2003. “Using Individualised Communication for Interviewing People With Intellectual Disability: A Case Study of User‐Centred Research.” Journal of Intellectual and Developmental Disability 28, no. 1: 5–23. 10.1080/136682503100008687.

[jar70132-bib-0019] Charitou, M. , E. Quayle , and A. Sutherland . 2021. “Supporting Adults With Intellectual Disabilities With Relationships and Sex: A Systematic Review and Thematic Synthesis of Qualitative Research With Staff.” Sexuality and Disability 39, no. 1: 113–146. 10.1007/s11195-020-09646-z.

[jar70132-bib-0020] Chen, Y. , M. Xia , and S. Dunne . 2024. “Romantic Love Is Not Only “Romantic”: A Grounded Theory Study on Love in Romantic Relationships.” Journal of Psychology 158, no. 1: 64–83. 10.1080/00223980.2024.2305442. 38285480

[jar70132-bib-0021] Cleaver, S. , H. Ouellette‐Kuntz , and A. Sakar . 2010. “Participation in Intellectual Disability Research: A Review of 20 Years of Studies.” Journal of Intellectual Disability Research 54, no. 3: 187–193. 10.1111/j.1365-2788.2010.01256.x. 20146739

[jar70132-bib-0022] Correa, A. B. , Á. Castro , and J. R. Barrada . 2022. “Attitudes Towards the Sexuality of Adults With Intellectual Disabilities: A Systematic Review.” Sexuality and Disability 40, no. 2: 261–297. 10.1007/s11195-021-09719-7.

[jar70132-bib-0023] Craft, A. 1994. Practice Issues in Sexuality and Learning Disabilities. Routledge.

[jar70132-bib-0024] Critical Appraisal Skills Programme (CASP) . 2013. “CASP Checklist: Systematic Reviews With Meta‐Analysis of Observational Studies.” https://casp‐uk.net/casp‐checklists/CASP‐checklist‐systematic‐reviews‐observational‐studies‐checklist‐2024.pdf.

[jar70132-bib-0026] Department of Health . 2001. “Valuing People: A New Strategy for Learning Disability for the 21st Century.” The Stationery Office. https://assets.publishing.service.gov.uk/media/5a7b854740f0b62826a041b9/5086.pdf.

[jar70132-bib-0025] Department of Health . 2009. “Valuing People Now: A New Three‐Year Strategy for People With Learning Disabilities.” The Stationery Office. https://lx.iriss.org.uk/sites/default/files/resources/Valuing%20people%20now.pdf.

[jar70132-bib-0027] Dew, A. , and K. M. Boydell . 2017. “Knowledge Translation: Bridging the Disability Research‐To‐Practice Gap.” Research and Practice in Intellectual and Developmental Disabilities 4, no. 2: 142–157. 10.1007/s11195-021-09719-7.

[jar70132-bib-0028] Dinwoodie, R. , B. Greenhill , and A. Cookson . 2020. ““Them Two Things Are What Collide Together”: Understanding the Sexual Identity Experiences of Lesbian, Gay, Bisexual and Trans People Labelled With Intellectual Disability.” Journal of Applied Research in Intellectual Disabilities 33, no. 1: 3–16. 10.1111/jar.12252. 27538684

[jar70132-bib-0029] Edgerton, R. 1979. Mental Retardation. Open Books.

[jar70132-bib-0030] English, B. , A. Tickle , and R. dasNair . 2018. “Views and Experiences of People With Intellectual Disabilities Regarding Intimate Relationships: A Qualitative Metasynthesis.” Sexuality and Disability 36, no. 2: 149–173. 10.1007/s11195-017-9502-z.

[jar70132-bib-0031] Fish, R. 2016. ““They've Said I'm Vulnerable With Men”: Doing Sexuality on Locked Wards.” Sexualities 19, no. 5–6: 641–658. 10.1177/1363460715620574.

[jar70132-bib-0032] Forrester‐Jones, R. , J. Beecham , A. Randall , et al. 2021. “The Impact of Austerity Measures on People With Intellectual Disabilities in England.” Journal of Long‐Term Care: 241–255. 10.31389/jltc.59.

[jar70132-bib-0033] Forrester‐Jones, R. , J. Carpenter , P. Cambridge , et al. 2002. “The Quality of Life of People 12 Years After Resettlement From Long‐Stay Hospitals: Users' Views on Their Living Environment, Daily Activities and Future Aspirations.” Disability & Society 17, no. 7: 741–758. 10.1080/0968759021000068469.

[jar70132-bib-0034] Forrester‐Jones, R. , J. Dixon , and B. Jaynes . 2023. “Exploring Romantic Need as Part of Mental Health Social Care Practice.” Disability & Society 39, no. 10: 2611–2633. 10.1080/09687599.2023.2222900.

[jar70132-bib-0035] Forrester‐Jones, R. , S. Jones , S. Heason , and M. Di'Terlizzi . 2004. “Supported Employment: A Route to Social Networks.” Journal of Applied Research in Intellectual Disabilities 17, no. 3: 199–208. 10.1111/j.1468-3148.2004.00199.x.

[jar70132-bib-0036] Forrester‐Jones, R. , and O. Raji . 2025. “Spiritual Assessment in Intellectual Disability.” In Spiritual Assessment in Healthcare: A Resource Guide, edited by L. Ross and W. McSherry , 1–19. Springer. 10.1007/978-3-031-78575-7_14.

[jar70132-bib-0037] Forrester‐Jones, R. V. E. , and G. Grant . 1997. Resettlement From Large Psychiatric Hospital to Small Community Residence: One Step to Freedom? Ashgate Publishing Group.

[jar70132-bib-0039] Gomez, M. T. 2012. “The S Words: Sexuality, Sensuality, Sexual Expression, and People With Intellectual Disability.” Sexuality and Disability 30, no. 4: 237–245. 10.1007/s11195-011-9250-4.

[jar70132-bib-0040] Heyman, B. 1995. “Sexuality as a Perceived Hazard in the Lives of Adults With Learning Difficulties.” Disability & Society 10, no. 2: 139–156.

[jar70132-bib-0041] Hollomotz, A. , and The Speakup Committee . 2009. ““May We Please Have Sex Tonight?” – People With Learning Difficulties Pursuing Privacy in Residential Group Settings.” British Journal of Learning Disabilities 37, no. 2: 91–97. 10.1111/j.1468-3156.2008.00512.x.

[jar70132-bib-0042] Human Rights Act . 1998. “c. 42. 1998.” https://www.legislation.gov.uk/ukpga/1998/42/contents/enacted.

[jar70132-bib-0043] Jacobs, P. , K. Watchman , H. Wilkinson , L. Hoyle , and L. McGinley . 2023. “Experiences of People With Intellectual Disability and Dementia: A Systematic Review.” Journal of Applied Research in Intellectual Disabilities 36, no. 2: 241–258. 10.1111/jar.13063. 36562340 PMC10107172

[jar70132-bib-0044] * Lafferty, A. , R. McConkey , and L. Taggart . 2013. “Beyond Friendship: The Nature and Meaning of Close Personal Relationships as Perceived by People With Learning Disabilities.” Disability & Society 28, no. 8: 1074–1088. 10.1080/09687599.2012.758030.

[jar70132-bib-0045] Landman, R. 1994. “Making Sex Safer for People With Learning Disabilities.” Nursing Times 90, no. 28: 35–37. 8065959

[jar70132-bib-0069] Määttä, K. 2011. “The Fascination of Love Never Fades – How do the Elderly Describe Their Experiences of Falling in Love.” International Review of Social Sciences and Humanities 2, no. 1: 14–25.

[jar70132-bib-0046] Majumder, S. , T. Mondal , and M. J. Deen . 2017. “Wearable Sensors for Remote Health Monitoring.” Sensors (Basel) 17, no. 1: 130. 10.3390/s17010130. 28085085 PMC5298703

[jar70132-bib-0047] Malli, M. A. , and R. Forrester‐Jones . 2025. Tourette's Syndrome, Stigma, and Society's Jests. Springer Nature. https://link.springer.com/book/10.1007/978‐3‐031‐83368‐7.

[jar70132-bib-0048] Mattila, J. , K. Määttä , and S. Uusiautti . 2016. “Everyone Needs Love – An Interview Study About Perceptions of Love in People With Intellectual Disability (ID).” International Journal of Adolescence and Youth 22, no. 3: 296–307. 10.1080/02673843.2016.1167749.

[jar70132-bib-0051] McCarthy, M. 1999. Sexuality and Women With Learning Disabilities. Jessica Kingsley Publishers.

[jar70132-bib-0052] McCarthy, M. 2014. “Women With Intellectual Disability: Their Sexual Lives in the 21st Century.” Journal of Intellectual & Developmental Disability 39, no. 2: 124–131. 10.3109/13668250.2014.894963.

[jar70132-bib-0049] * McCarthy, M. , C. Bates , N. Elson , S. Hunt , K. Milne‐Skillman , and R. Forrester‐Jones . 2022. ““Love Makes Me Feel Good Inside and My Heart is Fixed”: What Adults With Intellectual Disabilities Have to Say About Love and Relationships.” Journal of Applied Research in Intellectual Disabilities 35, no. 4: 955–965. 10.1111/jar.12893. 34033223

[jar70132-bib-0050] McCarthy, M. , K. Milne‐Skillman , N. Elson , C. Bates , R. Forrester‐Jones , and S. Hunt . 2020. “Making Connections and Building Confidence: A Study of Specialist Dating Agencies for People With Intellectual Disabilities.” Sexuality and Disability 38: 3–18. 10.1007/s11195-020-09619-2.

[jar70132-bib-0053] Murphy, G. H. , and A. O'Callaghan . 2004. “Capacity of Adults With Intellectual Disabilities to Consent to Sexual Relationships.” Psychological Medicine 34, no. 7: 1347–1357. 10.1017/S0033291704001941. 15697061

[jar70132-bib-0054] Noblit, G. W. , and R. D. Hare . 1988. Meta‐Ethnography (Qualitative Research Methods). SAGE Publications, Inc.

[jar70132-bib-0055] O'Brolcháin, F. , and B. Gordijn . 2019. “Privacy Challenges in Smart Homes for People With Dementia and People With Intellectual Disabilities.” Ethics and Information Technology 21: 253–265. 10.1007/s10676-019-09507-0.

[jar70132-bib-0056] Oliver, M. 2013. “The Social Model of Disability: Thirty Years On.” Disability & Society 28, no. 7: 1024–1026. 10.1080/09687599.2013.818773.

[jar70132-bib-0057] Page, M. J. , J. E. McKenzie , P. M. Bossuyt , et al. 2021. “The PRISMA 2020 Statement: An Updated Guideline for Reporting Systematic Reviews.” BMJ 372, no. 71: 1–9. 10.1136/bmj.n71. PMC8005924 33782057

[jar70132-bib-0058] Parchomiuk, M. 2013. “Model of Intellectual Disability and the Relationship of Attitudes Towards the Sexuality of Persons With an Intellectual Disability.” Sexuality and Disability 31: 125–139. 10.1007/s11195-012-9285-1. 23704800 PMC3659271

[jar70132-bib-0059] Pérez‐Curiel, P. , E. Vicente , M. L. Morán , and L. E. Gómez . 2023. “The Right to Sexuality, Reproductive Health, and to Found a Family for People With Intellectual Disability: A Systematic Review.” International Journal of Environmental Research and Public Health 20, no. 2: 1587. 10.3390/ijerph20021587. 36674341 PMC9864803

[jar70132-bib-0060] * Rushbrooke, E. , C. Murray , and S. Townsend . 2014. “The Experiences of Intimate Relationships by People With Intellectual Disabilities: A Qualitative Study.” Journal of Applied Research in Intellectual Disabilities 27, no. 6: 531–541. 10.1111/jar.12091. 24478268

[jar70132-bib-0068] Swain, J. , and S. French . 2000. “Towards and Affirmation Model of Disability.” Disability & Society 15, no. 4: 569–582. 10.1080/09687590050058189.

[jar70132-bib-0061] Thomas, J. , and A. Harden . 2008. “Methods for the Thematic Synthesis of Qualitative Research in Systematic Reviews.” BMC Medical Research Methodology 8, no. 45: 1–10. 10.1186/1471-2288-8-45. 18616818 PMC2478656

[jar70132-bib-0062] Thompson, D. , and H. Brown . 1997. “Men With Intellectual Disabilities Who Sexually Abuse: A Review of the Literature.” Journal of Intellectual Disabilities 10, no. 2: 140–158. 10.1111/j.1468-3148.1997.tb00014.x.

[jar70132-bib-0063] Thompson, S. A. , M. Bryson , and S. de Castell . 2001. “Prospects for Identity Formation for Lesbian, Gay, or Bisexual Persons With Developmental Disabilities.” International Journal of Disability, Development and Education 48, no. 1: 53–65. 10.1080/10349120120036305.

[jar70132-bib-0067] Ward, K. M. , R. L. Bosek , and E. L. Trimble . 2010. “Romantic Relationships and Interpersonal Violence Among Adults With Developmental Disabilities.” Intellectual and Developmental Disabilities 48, no. 2: 89–98. 10.1352/1934-9556-48.2.89. 20597743

[jar70132-bib-0064] Watchman, K. , P. Jacobs , L. Boustead , et al. 2024. ““How Will We Cope?” Couples With Intellectual Disability Where One Partner Has a Diagnosis of Dementia.” Gerontologist 64, no. 6: 1–10. 10.1093/geront/gnae030. PMC11127106 38505929

[jar70132-bib-0065] * White, E. , and R. Barnitt . 2000. “Empowered or Discouraged? A Study of People With Learning Disabilities and Their Experience of Engaging in Intimate Relationships.” British Journal of Occupational Therapy 63, no. 6: 270–276. 10.1177/030802260006300605.

[jar70132-bib-0066] * White, P. , and R. Forrester‐Jones . 2020. “Valuing e‐Inclusion: Social Media and the Social Networks of Adolescents With Intellectual Disability.” Journal of Intellectual Disabilities 24, no. 3: 381–397. 10.1177/1744629518821240. 30616492

